# Quantification of 4-Methylimidazol in NMRI Mice Plasma and Cerebrospinal Fluid (CSF) by Using Liquid Chromatography Tandem Mass Spectrometry

**DOI:** 10.22037/ijpr.2020.112406.13740

**Published:** 2020

**Authors:** Fereshteh Mehri, Firouzeh Nazari, Zohreh Fasihi, Farzad Kobarfard

**Affiliations:** a *Nutrition Health Research Center, Hamadan University of Medical Sciences, Hamadan, Iran. *; b *Food and Drug Organization, Iran University of Medical Sciences, Tehran, Iran. *; c *Department of Medicinal Chemistry, School of Pharmacy, Shahid Beheshti University of Medical Sciences, Tehran, Iran. *; d *Phytochemistry Research Center, Shahid Beheshti University of Medical Sciences, Tehran, Iran.*

**Keywords:** 4-Methylimidazole, NMRI mice, Plasma, Cerebrospinal fluid, LC-MS/MS

## Abstract

A sensitive method using ion-pair extraction was developed by liquid chromatography tandem mass spectrometry (LC–MS/MS) for measurement of 4-methylimidazole (4-MI) in NMRI mice plasma and cerebrospinal fluid (CSF). Detection was done by electrospray positive ionization mass spectrometry in the multiple-reaction monitoring (MRM) mode. The validation method was applied to quantification of 4-MI in plasma and CSF samples using oral doses of 100, 200, and 300 mg/kg in NMRI mice. The efficiency of the method was evaluated in terms of linearity (R ^2^> 0.99), recovery (98–107%, 3 levels) and precision (8–10%, 3 levels, n = 6). Limit of detection (LOD) and limit of quantification (LOQ) were 25 ng/mL and 50 ng/mL, respectively. The results obtained showed that the exposure to oral doses of 4-MI in mice makes different concentrations in plasma and CSF and causes significant changes in mice. This study was the first report for determination of 4-MI in plasma and CSF samples in mice. Our results suggest that LC-MS/MS-based on ion-pair extraction is a robust method with high detection ability for measurement of 4-MI in plasma and CSF samples. Therefore, the developed method can be useful for evaluation and monitoring of imidazole derivatives in biological samples.

## Introduction

Considering the important role of foods and beverages in human health, the concern about many contaminants, additives and toxins in high consumption foods and their effects on human health has been the subjective for many studies since the past several decades (1, 2). 4-Methylimidazole (4-MI) is the by-product of Millard reaction between carbohydrates and amine-containing compounds which forms as an impurity during the manufacturing Class III and Class IV Caramel Colorings (3). It has high bioavailability and quickly distributes in intestine, liver, blood, and kidney (4). This group of color additives are widely used in different beverages and foods including cola, coffee, beer, as well as other products like soy sauce and baked goods (5). 4-MI is classified as possibly carcinogenic to humans (group 2B) and exposure to the concentration higher than 250 mg/kg 4-MI in Class III and Class IV caramel may lead to increasing tumors, different cancers and functional disorders in animals (6, 7). It has been shown in many toxicological studies that 4-MI had different toxic effects on organs such as CNS, kidney, and lung and it may be hazardous to human health (8). Chan *et al*., in 2008 showed that the exposure to 4-MI may lead to the risk of tremors, ataxia and anemia in F344/N rat and the risk of lung tumors in B6C3F1 mice (9). In other studies it has been showed that 4-MI has neurobehavioral toxicity in mother and developmental impairments in the offspring and could induce alveolar/bronchiolar adenoma and carcinoma in male and female mic (10-12). According to the U.S. NTP report, 4-MI has toxic effects on CNS and can elicit neurological signs and forms potent convulsion in mice, chicks, rabbits, and cattle (13, 14). There are different analytical methods to determine the concentration of 4-MI in most foods and beverages. The most commonly used methods are including thin-layer chromatography (TLC), high performance liquid chromatography (HPLC), GC–MS, and capillary electrophoresis (15, 16). Recently, liquid chromatography, coupled with tandem mass spectrometry (LC-MS/MS) because of need to very small amounts of samples, high sensitivity and specificity, ability to identify and analysis in low concentration, and also high potency and accuracy for quantification of many targets in a single run has been widely used as an interesting alternative method in laboratory investigations (17) .Considering the 4-MI apply in the manufacturing of dyes and pigments, agricultural chemicals, and rubber and also penetrability of it into the brain tissue (18). On one hand and since there is not a fast and reliable method for the extraction and quantification of 4-MI in biological samples on the other hand, determination of this contamination in biological samples is necessary. Therefore, this research was conducted to fill the gap in the literature with the aim of determining 4-MI concentration in plasma and CSF by using LC/MS/MS. The results of this study can be used in future studies to better understand the mechanisms involved in toxicity of 4-MI.

## Experimental

*Reagents*

4-Methylimidazole (C4H6N2: MW; 82, purity 99%, West Chester, PA; USA), methanol and formic acid were purchased from Sigma-Aldrich. Phosphate buffer 1 M composed of potassium dihydrogen phosphate; dipotassium phosphate (West Chester, PA, USA) were purchased from (West Chester, PA, USA). 2-Chloropyridine as internal standard (IS) was prepared from (Merck Darmstadt, Germany).Bis-2-ethylhexylphosphate (BEHPA; purity 98%), chloroform (purity 99%) and phosphoric acid (purity 99%) for extraction phase was purchased from Sigma-Aldrich. All other reagents were analytical grade.

*Ethical procedures and sample collection*

All the animal experiments were done according to procedures approved by the guidelines of Animal handling of Shahid Beheshti University of Medical Sciences, Tehran, Iran. In the present study 70 male mice (18-25 g and 4-6 week age) were exposed to 4-MI in three group100 mg/kg (N = 20), 200 mg/kg (N = 20), 300 mg/kg (N = 20) and the control group (N = 10) respectively for 14 days. In the end, on the fourteenth day, the mice were anesthetized with ketamine and xylazine injection and the blood and CSF samples were collected from cardiac puncture and cisternamagna, respectively. In order to separate plasma and blood, the samples were treated in heparinized tubes. The control animals received an isovolumetric injection of sterile saline. 

*Standards solution*

In the beginning, 4-MI was dissolved in deionized (DI) water to provide 1000 ng/mL primary stock solutions. Working standard solutions were prepared from the stock solution at a concentration10 µg/mL. The stock solution of 2-chloroprydine as internal standard (IS) was prepared in DI water with a concentration of 10 µg/mL for constructing the calibration curve. Calibration standards were prepared at 5 concentrations: 50 ng/mL, 100 ng/mL, 200 ng/mL, 500 ng/mL, and 1000 ng/mL with IS spiked at 100 ng/mL for each level. Aliquots of each working solution were diluted 50 fold with 4-MI-free plasma or CSF to obtain five points to draw calibration standards (CS) curve and recovery samples. All the solutions were stored at 4 °C until analysis.

*Sample preparation*

 The extraction procedure is adapted from the method published by Thomsen *et al*., (1981). Four hundred µL of plasma was pipetted into a screw-capped glass tube and 2.5 mL of a 0.2 M potassium phosphate buffer (pH 6.7.0) was added to it. The tube was capped and vortex-mixed for 25s. Then 4 mL of 0.1 M bis2-ethylhexyl hydrogen phosphate (BEHPA) in chloroform were added to this mix, and the tube was vortex-mixed again for 30s to extract 4MI. After centrifugation at 3500 g (RPM) for 5 min at ambient temperature, the aqueous phase (top layer) was removed by aspiration and 3 mL of the chloroform phase (bottom layer) were transferred to a new centrifuge tube containing 3 mL of 0.1 M phosphoric acid. The tube was vortex-mixed again for 30s and 4MI re-extracted into the aqueous phase. After centrifugation at 3500 g for 5 min, the aqueous phase (top layer) was ready for injection. In the case of CSF samples: 200 µL of CSF from mice was pipetted into screw-capped glass tubes, 2 mL of 0.2 M phosphate buffer (PH 6.0) was added to it. The extraction of 4-MI from CSF samples was performed as previously described for plasma samples. Finally, 100 µL of plasma and CSF samples obtained from the extraction process were used in the later steps for injection in LC-MS-MS (19).

*Chromatographic and mass spectrometric conditions*

Detection and quantification were performed using a 6410 triple quadrupole Agilent LC-MS/MS system (Agilent Santa Clara, CA, USA) with a 1200 series HPLC system, equipped with an electrospray ionization (ESI) source. Chromatographic separation was performed on a Gemini C18 column (150x, 4.6 mm, 3 µm) (Phenomenex, Torrance, CA). Flow rate and injection volumes were 0.5 mL /min and 20 µL, respectively. The mobile phase was 25% of 0.2 M phosphate buffer with 0.1% formic acid (solvent A) and 75% methanol with 0.1% formic acid (solvent B) in isocratic mode. The total time of analysis was 15 min. ESI/MS/MS was performed in the scheduled multiple reactions monitoring (MRM) positive mode with the following settings: capillary temperature of 250 °C and spray voltage of 4500 V. The desolation gas was nitrogen at 60 psi. The transitions and collision energies for the analyst and internal standard are given in Table 1.

*Method validation*

Since the plasma is a more complex matrix than CSF due to the presence of proteins, glucose and cholesterol content, a validation study was performed on plasma samples according to FDA guidelines (20). The performance of the method was assessed to obtain the limit of detection (LOD), the limit of quantification (LOQ), the linearity and the precision and recovery. The linearity was calculated by three separate runs of the spiked blank sample at five known concentrations between 50 ng/mL to 1000 ng/mL. LOD and LOQ were calculated as signal-to-noise ratio at 3:1 and 10:1, respectively. To determine the recovery and precision of extraction, 

three levels of 4-MI concentrate, such as 75 ng/mL, 350 ng/mL, and 750 ng/mL were spiked to blank plasma and then the treated samples according to the procedure were described in sample preparation.

*Statistical analysis*

The obtained results were entered into the SPSS-16 software and the analysis of variance test (ANOVA) and Tukey *Post-hoc *test was used to evaluate the relationship between the studied variables. The data have been reported based on mean ± SE and *p* < 0.05 was considered statistically significant.

## Results and Discussion

*Validation of method*

Because of low evaporation and high polarity of imidazole derivatives, extraction efficiency with the most organic solvents is low (16, 21, 22). On the other hand, 4-MI is a low ionized compound and cannot efficiently be extracted into immiscible water solvents. Therefore, in order to increase its solubility into the organic phase, an ion-pair system was necessary which BEHPA was used in this study. This compound increased the extraction efficiency of 4-MI in chloroform in the extraction process (19) effect of pH in the extraction process was very important. Improper pH will decrease the extraction efficiency for 4-MI. Different pH values from 5 to 7 were tried and the best result was obtained with potassium phosphate buffer of pH 6.70 and pH 6.00 for plasma and CSF samples, respectively. The results were in agreement with the report obtained by Thomsen *et al., *(1981) in sheep plasma (19). Different columns such as SeQuant ZIC HILIC, porous graphite carbon (Phenomenex Hypercarb 100 mm, 2.1 mm, 5 µm), and Gemini RP 18 have been used and reported for separation of 4-MI in caramelized drinks (23). Our findings showed the best separation achieved by Gemini RP 18 column along with short run time and high capacity. The selection of the mobile phase was necessary for the measurement of the accurate concentration of 4-MI in biological samples. Methanol and phosphate buffer (75; 25) along with 0.1% formic acid was used to increase resolution. Due to the wide use of 4MI in industry, its presence in different kinds of food products, and the lack of determination procedures in the biological sample, the method developed in the present study could be useful for future studies. This method could also be applied for neurotoxicity studies of 4-MI in animals and humans.

*Occurrence of 4-MI in plasma and CSF sample*

**Figure 1:**
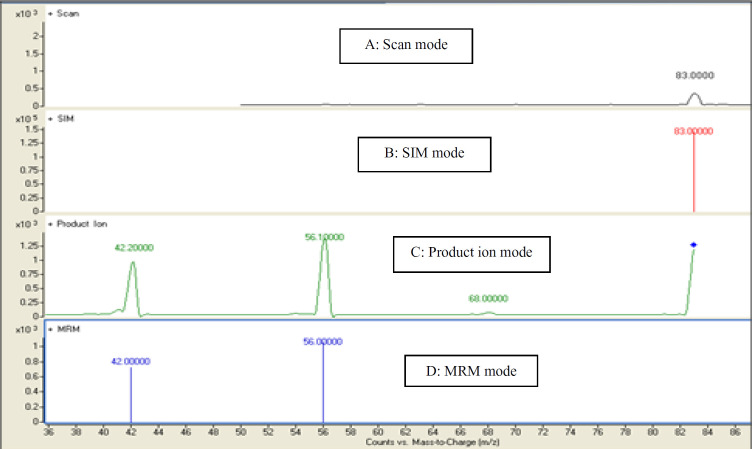
Representative chromatograms of 4-MI (m/z 83.59) in (A) Scan mode (B) SIM mode (C) product ion mode (D) MRM mode

**Figure 2:**
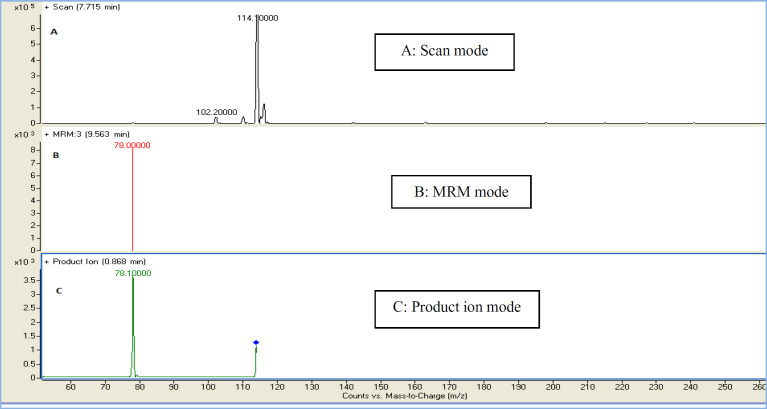
Representative chromatograms of 2-chloroprydine (I.S) (m/z 144.78) in (A) Scan mode (B) MRM mode (C) product Ion mode

**Figure 3:**
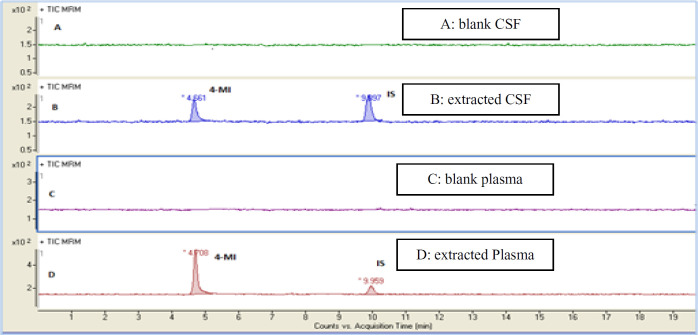
Representative MRM chromatograms of (4-MI m/z 83.59) and (2-chloroprydine I.S. m/z 144.78) in control group: (A) blank CSF and (C) blank plasma, in treatment group after oral administration with 4-MI at a single dose of 200 mg/kg to mice; (B) extracted CSF; (D) extracted Plasma

 In the present study, a sensitive and precise method using LC/MS/MS for the determination of 4-MI in plasma and CSF samples was developed. Recent developments in MS technology have been widely used for food sample analysis. In this study calibration curve and linearity of the method were assessed using the matrix-matched calibration solutions at five concentrations (50 ng/mL, 100 ng/mL, 200 ng/mL, 500 ng/mL, and and1000 ng/mL) as above described. Calibration curves were constructed by plotting the 4-MI/I.S areas ratio of peaks against the concentration values. The summarized findings in Table 2 showed that the obtained data had good linearity with correlation coefficients higher than 0.998. LOD and LOQ for both plasma and CSF samples were 25 ng/mL and 50 ng/mL respectively. Recovery and precision were determined on spiked samples of plasma containing 4-MI at three concentration levels. As shown in Table 3 the average of recoveries was ranging from 98 to 107%. These results were acceptable in the range set by Codex Alimentations Commission (2002) (24). The chromatograms obtained in scan mode, selected ion monitoring (SIM) mode, product ion mode and multiple reaction monitoring mode (MRM) of 4-MI (m/z: 83→42; 83→56) and 2-chloroprydine (IS): (m/z 114→78) in plasma and CSF samples at concentration of 100 ng/mL are shown in Figures 1 and 2, respectively. Retention times for 4-MI and IS were 4.5 and 9.8 min respectively. The results obtained from the exposure of the mice to different oral doses of 4-MI and their concentrations in the plasma and CSF samples are shown in Table 4. In the mice which had received 100 to 300 mg/kg oral doses of 4-MI, the obtain plasma concentration was between 630 to 2600 ng/mL. On the other hand, the concentration of 4-MI in CSF samples of the mice which had received 200 and 300 mg/kg oral dose of 4-MI obtained between 50 to 90 ng/mL. However, in the mice which had received an oral dose of 100 mg/kg, the concentrations were lower than LOD. The smallest levels of 4-MI obtained in plasma (25 ng/mL) was much less than the 4000 ng/mL reported in sheep plasma by Karangwa *et al*., (1990) (25). The results obtained in this study showed that the plasma samples had a high concentration of 4-MI in comparison with CSF samples. The reason for this lower concentration of 4-MI in CSF sample can be attributed to the protective role of the blood-brain barrier (BBB) that controls the passage of selected substances into the brain while transporting toxic products back into circulation (26). Figure 3 shows the chromatograms of blank and extracted samples of plasma and CSF. The chromatograms of blank and spiked samples of plasma and CSF imply there weren’t interfering peaks from endogenous substances. Previous surveys about the neurotoxicity of 4-MI showed that this compound could distribute into the brain and affect the cerebral GABA levels and induce inhibitors of the cerebral glutamic acid decarboxylase (GAD) activity (27) which ultimately may cause sudden episodes of agitated confusion, excitability, tremors, and convulsions (28). Also, our previous study indicated that 4-MI has a toxic effect on mice brain mitochondria (10). According to the results of the present study, it is concluded that the LC-MS/MS method had satisfactory specificity, precision, accuracy, and high recovery for the determination of 4-MI concentration in plasma and CSF.

**Table 1:** MRM transitions and collision energies for 4-Methylimidazol and 2-chloropryidine

**dwell time**	**Collision energy (eV)**	**Product ion (m/z)**	**Precursor ion (m/z)**	**Mass (Da)**	**Compound**
100	20	42	83	82.11	4-Methylimidazol
100	20	56	83	82.11	4-Methylimidazol
80	40	78	114	113.54	2-chloroprydine

**Table 2:** Chromatographic retention data, regression analysis, limits of detection and limit of quantification for 4-MI.

Concentration range ng/mL: n = 18	Retention time (min)	Regression Equation	R2	LOD (ng/mL)	LOQ (ng/mL)
25-1000	4.9	Y = 0.003x-0.018	0.999	25	50

Notes: LOD, the limit of detection; LOQ, the limit of quantification.

**Table 3:** Recovery, (Intra- and inter-assay) and precision values for determination of 4-MI

	**Inter-assay**			**Intra-assay**		
Concentration(ng/mL)	75	250	750	75	250	750
Mean	0.20	1.03	2.66	0.21	1.06	2.62
SD	0.01	0.03	0.15	0.027	0.06	0.10
CV (%)	0.08	0.03	0.05	0.12	0.05	0.03
RE% (mean)	101.11	98.00	107.73	104.24	99.00	109.11

Notes: SD, standard deviation; CV, coefficient of variation, RE, recovery. Intra-assay experiments were performed by preparing 6 replicates of plasma samples. Inter-assay experiments were performed by repeatedly analyzing samples in different runs over 6 days.

**Table 4:** Plasma and CSF concentration of 4-MI after oral administration with different dosing in mic

**Mean concentration** **ng/mL**	**Number samples obtained in each group**	**Number of mice in each group**	**Oral dose**	**Location**
0.63 ± 0.1	14	20	100 mg/kg	Plasma
1.5 ± 0.6	16	20	200 mg/kg	
2.6 ± 0.9	17	20	300 mg/kg	
Lower of LOD	ND	20	100 mg/kg	CSF
0.05 ± 0.01	16	20	200 mg/kg	
.0.09 ± 0.04	16	20	300 mg/kg	
ND^a^	ND	10	saline	control

1^a^ N.D: not detected.

## Conclusion

This study was the first reported sample for the determination of 4-MI in Plasma and CSF sample in mice using a modified method. The proposed method was based on ion-pair extraction using BEHPA and the quantification by LC–MS/MS. The method was fully validated in terms of the sensitivity, linear range, precision/accuracy and recovery and has been successfully applied to the analysis of the real samples of plasma and CSF in mice. The best results were obtained in pH 6.7.00 and PH 6.00 for plasma and CSF samples, respectively. The mobile phase was composed of methanol and phosphate buffer (75; 25) along with with0.1% formic acid, and the column was Gemini RP 18. The results obtained in this study showed excellent recoveries (98-107%) and precision (SD < 2%) in samples. Our study showed that exposure to different doses of 4-MI in mice makes various concentrations in plasma and CSF. Because of high sensitivity and accuracy of LC/MS/MS, this method is suitable for toxicity assessment of 4-MI in animal and human subjects and also the results of this study can be applied for future investigation about the possible mechanism and assessment of 4-MI in neurotoxicity in animals and humans.
